# Pregnancy in patients with tuberculosis: a TBNET cross-sectional survey

**DOI:** 10.1186/s12884-016-1096-4

**Published:** 2016-10-12

**Authors:** Graham H. Bothamley, Cordula Ehlers, Irina Salonka, Alena Skrahina, Angels Orcau, Luigi R. Codecasa, Maurizio Ferrarese, Dragica Pesut, Ivan Solovic, Andrii Dudnyk, Luis Anibarro, Claudia Denkinger, Lorenzo Guglielmetti, Inge Muylle, Marco Confalonieri

**Affiliations:** 1 Department of Respiratory Medicine, Homerton University Hospital, London, E9 6SR UK; 2 TBNET Office, Centre for Research-Borstel, Borstel, Germany; 3 Republican Research and Practical Centre for Pulmonology and TB, Minsk, Belarus; 4 Agència de Salut Pública de Barcelona, Barcelona, Spain; 5 Regional TB Reference Centre, Villa Marelli Institute, Niguarda Ca’Granda Hospital, Milan, Italy; 6 University of Belgrade School of Medicine, Belgrade, Serbia; 7 National Institute for Tuberculosis, Pulmonary Disease and Thoracic Surgery Vyšné Hágy, Ruzomberok, Slovakia; 8 National Pirogov Memorial Medical University, Vinnytsia, Ukraine; 9 Hospitalario Universitario de Pontevedra, Pontevedra, Spain; 10 Beth Israel Deaconess Medical Center, Boston, USA; 11 Department of Infectious Diseases, University of Verona, Verona, Italy; 12 UMC St. Pieter - CHU St. Pierre, Brussels, Belgium; 13 University Hospital of Cattinara, Trieste, Italy

**Keywords:** Tuberculosis, Pregnancy, TBNET, Diagnosis, Postpartum

## Abstract

**Background:**

Objectives: To determine whether the incidence of tuberculosis with pregnancy is more common than would be expected from the crude birth rate; to see whether there is significant delay in the diagnosis of tuberculosis during pregnancy.

**Method:**

Design: A cross-sectional survey. Setting: 13 tuberculosis clinics within different European countries and the USA. Population/sample: All patients with tuberculosis seen at these clinics for a period > 1 year. Instrument: Questionnaire survey based on continuous data collection. Main outcome measures: number and proportion of women with tuberculosis who were pregnant; timing of diagnosis in relation to pregnancy, including those who were pregnant or delivered in the 3 months prior to the diagnosis of TB and those who developed TB within 3 months after delivery.

**Results:**

Pregnancy occurred in 224 (1.5 %) of 15,217 TB patients and followed the expected rate predicted from the crude birth rate for the clinic populations. TB was diagnosed more commonly in the 3 months after delivery (*n* = 103) than during pregnancy (*n* = 68; *χ* ^2^ = 25.1, *P* < 0.001).

**Conclusions:**

TB is diagnosed more frequently after delivery, despite variations in local TB incidence and healthcare systems.

## Background

Tuberculosis most commonly affects women during their reproductive years [1]. Pregnancy during the treatment of tuberculosis was formerly more common due to the interaction between the oral contraceptive pill and rifampicin, such that the former was no longer effective [2]. In pregnancy there is also a shift from cell-mediated (Th1) immunity, which protects against tuberculosis [3], to antibody-mediated (Th2) immunity [4–6], making reactivation of latent tuberculosis and susceptibility to recent infection progressing to active disease more likely.

The diagnosis of tuberculosis is often delayed as many of the symptoms are non-specific and may be present during normal pregnancy, e.g. tiredness, feeling hot and sweating at night [7]. Exposure of the fetus to x-rays from chest radiography raises concerns in pregnant women and health care workers [8] and so the usual process of diagnosis for those with a cough may be delayed. The World Health Organization recommends screening for tuberculosis in those with HIV co-infection and those with symptoms of tuberculosis using two-three sputum smears stained for tubercle bacilli and/or PCR tests for tuberculosis such as Xpert MTB/RIF [9]. However, the sputum smear is less frequently positive during pregnancy than in others with tuberculosis [10]; the value of PCR tests is in the early stages of evaluation [11]. Extrapulmonary tuberculosis requires tissue sampling and culture and screening pregnant women with symptoms is often provoked by a positive tuberculin skin test or interferon-gamma release assay [12]. Estimates have suggested that 26 % of all preventable deaths in pregnancy worldwide are directly attributable to tuberculosis [13] and children are especially susceptible to forms of tuberculosis with a high mortality [14]. Late diagnosis of active tuberculosis in pregnant women has significant cost implications. The possible transmission of tuberculosis to the baby and also to other mothers and their children requires extensive contact tracing and constitutes a “serious untoward incident”.

Our aim was to determine whether tuberculosis was in fact more common in pregnancy and whether the developed health care systems in Europe and the United States were still associated with a diagnostic delay of tuberculosis in pregnant women.

## Methods

### Study design

Cross-sectional survey.

#### Setting

The Tuberculosis Network European Trialsgroup (TBNET; http://www.tb-net.org/) is made up of >650 physicians who wish to participate in clinical trials in tuberculosis and who therefore aim to maintain accurate data regarding their patients [15]. A survey of TBNET members was conducted to review clinical records of those with tuberculosis from 1 September 2008 to 31 August 2013 if possible, or at least for a period of one year or more.

#### Participants

Participants were required to have recorded routinely whether patients were pregnant or not and, from contact tracing records, to be able to determine whether they were breastfeeding infants. Only clinics with at least one year of data and with >30 patients were included. Local ethical advice was sought; the study deemed an audit promoting good medical care.

### Variables

The data collected included the total number of patients with tuberculosis and the number who were pregnant during treatment for tuberculosis or within 3 months of delivering a child (mycobacterial culture may take up to 6 weeks to make the diagnosis of tuberculosis and hence the extended time period of 3 months rather than the standard 6 weeks postpartum period) or pregnant within 3 months of completing treatment for tuberculosis. The categories were such that if a pregnant woman decided not to continue with the pregnancy, the figures would still be included as “pregnant with tuberculosis”. Expected values were derived from the World Bank website containing 2010–2015 data for the crude birth rate [16].

#### Statistics

Participation was sought through an on-line questionnaire (SurveyMonkey.co.uk). Data were then re-entered on a standardized excel chart (Microsoft Office 2007). Data were checked again by asking for clarification of individual entries and at the stage of preparation of the Table. Where there were large numbers, e.g. Belarus, two authors were included to indicate the role of both in ascertaining integrity of the data. A *χ* ^2^-squared value was calculated using the formula (observed - expected)^2^/(expected) (GraphPad Software Inc. 2×2 contingency tables).

## Results

**Fig. 1:**
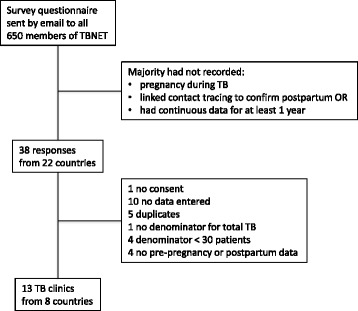
Flow chart of data collection

Thirteen sites provided data (Fig. 1), two clinics being in the same city of Milan. Numbers of patients with tuberculosis varied from 103 to 5,500 from data collected over a year (3 clinics), 3 years (3 clinics) or ≥5 years (7 clinics) (Table 1). The survey identified 224 who satisfied the criteria for inclusion in the study out of a denominator of 15,217 tuberculosis patients (1.5 %; median 0.7 %, range 0–3.1 %, with the Belgian clinic as an outlier at 11.1 %). The combined data showed that the number of pregnancies in those with tuberculosis was close to that predicted by the crude birth rate for the denominator (227; Table 1), but there were significant variations from country to country.

**Table 1:** Expected and actual numbers of pregnancies with concurrent TB

Country	No. of TB patients	Crude birth rate (per 1,000 per year)[16]	Expected number of pregnant TB patients^b^	During: TB diagnosed during pregnancy	Before: Pregnant within 3 months of completing TB treatment	After: TB diagnosed within 3 months after delivery	Predicted number of pregnant TB for 1.25 yrs	Total in study
Belarus	5500	13	54.9	15	16	6	89.4	37
Belgium	143	12	1.3	11	0	5	2.2	16
Italy (3 cities)								
A	1860			1	7	2		10
B	217			1	4	0		5
C	103			0	1	1		2
Sub-total	2180	9	15.1	2	12	3	24.5	17
Serbia	1353	9	9.4	2	0	1	15.3	3
Slovakia	1197	11	10.1	4	4	21	16.6	29
Spain (2 cities)								
A	1946			2	3	5		10
B	374			0	1	2		3
Sub-total	2320	10	17.8	2	4	7	29.0	13
Ukraine	500	11	4.2	3	10	35	6.9	48
United Kingdom	1774	18^a^	24.5	29	7	20	39.8	56
USA	250	13	2.5	0	0	5	3.3	5
Total	15,217	n/a	139.8	68	53	103	227.0	224

^a^Local birth rate (UK crude birth rate 12 per 1,000)

^b^ = no. of TB patients x crude birth rate x (280 days per pregnancy/365 days per year)

Sixty-eight had a diagnosis of tuberculosis during pregnancy (0.5 % all tuberculosis cases; 30 % of total included in the study, average 7.5 per calendar month (p.c.m.) for all pregnancies with tuberculosis) compared to 103 diagnosed with tuberculosis within 3 months of delivery (34.3 p.c.m.; *χ* ^2^ = 25.1, *P* < 0.001). The number of patients recorded as having become pregnant within 3 months of completing tuberculosis treatment (53/3, i.e. 17.7 p.c.m.) was also higher but did not differ significantly from the combined average per month for pregnancy and the 3-month period after delivery (171/12, i.e. 14.3 p.c.m., *χ* ^2^ = 0.8, *P* > 0.1). Tuberculosis was diagnosed more frequently during pregnancy in Belgium and the United Kingdom, and Ukraine less frequently than expected from the combined data (*χ* ^2^ = 6.3, 6.1 and 10.3, respectively, *P* <0.05). For individual countries, excluding those with less than 5 cases of tuberculosis diagnosed during pregnancy, again Belgium and the United Kingdom showed no significant difference in rate compared to the 3 months before and after pregnancy (all *χ* ^2^ ≤ 3.3, *P* > 0.05).

## Discussion

### Main findings

The most important finding is that tuberculosis is diagnosed less frequently during pregnancy than in the postpartum period. The rate of tuberculosis in pregnancy did not differ significantly when compared to the crude birth rate.

### Interpretation

There are several possible reasons for a late diagnosis of tuberculosis. Late booking for antenatal care amongst the population most likely to acquire tuberculosis may follow the inverse care law, whereby those in greatest need of medical care are least likely to access it [17, 18]. The population at risk of tuberculosis may have a poorer uptake of antenatal care and therefore be only susceptible to diagnosis at or shortly after delivery [10]. In a multicultural qualitative study close to the UK clinic, issues of ambivalence, lack of self worth and empowerment, common in patients with tuberculosis, have been recorded as reasons for delay in antenatal care [19].

The lower rate of diagnosis of tuberculosis during pregnancy compared to the 3 month postpartum period raises concerns. There may still be a reluctance to investigate pregnant women for tuberculosis, especially by performing a chest x-ray even though radiation doses are much reduced and the fetus can be shielded with a lead apron [4, 20]. A UK cohort study of 5.5 % of the total population also observed more tuberculosis notifications after pregnancy, documenting 22 diagnoses of tuberculosis during pregnancy and 22 in the 6 months post partum, rising to a peak at 90 days and falling thereafter [21]. These authors also noted that the postpartum increase in tuberculosis might be due to a delay in diagnosis.

An alternative reason for an increase in tuberculosis during the postpartum period might be that the immunological changes in pregnancy might allow latent tuberculosis infection to reactivate, a process which might take several weeks to pass from latent to subclinical disease and thence to active tuberculosis. In general, in pregnancy there is a shift from cell-mediated (Th1) immunity to antibody-mediated (Th2) immunity. Whilst the role of immunity in successful pregnancy is undoubtedly more complex, involving Treg cells with specificity for allogeneic antigens expressed by the fetus and NK cells [22, 23], HLA-G [24] and macrophages and dendritic cells [25], reduced levels of the pro-inflammatory cytokine IL-17 may also contribute to the likelihood of developing tuberculosis [26–28].

The burden of tuberculosis in pregnancy has been estimated from data available from the World Health Organization [29]. Using data for age and sex, birth rate and case notification by age and sex, the rate of tuberculosis was estimated at of 2.1 globally, 0.4 in the Americas and in Europe 0.6 per 1,000 pregnant women. Notably, the prevalence of tuberculosis was similar in the whole population to that in women aged 15–44 years. The predicted rate of pregnancy in women with tuberculosis would therefore be comparable to the crude birth rate x the number of days pregnant in a year (their estimate 280/365), i.e. 0.7–2.5 per 100 TB patients within the World Health Organization European Region.

Antenatal care is often the first occasion that women contact health services and thus has been suggested as a point at which verbal screening regarding symptoms of and risk factors for tuberculosis could occur in high risk populations [12, 30]. World Health Organization guidelines on screening for tuberculosis have emphasized those with human immunodeficiency virus infection and contacts of those with pulmonary tuberculosis, with consideration of migrants from high burden countries (tuberculosis incidence > 100 per 100,000) as well as prisoners, health care workers, homeless and illicit drug users [31]. Even in low tuberculosis incidence countries, screening for tuberculosis infection with an interferon-gamma release assay is considered cost effective [32].

#### Strengths and limitations

This is one of the largest surveys of pregnancy and tuberculosis to date (systematic reviews were identified from PubMed using the search terms “pregnancy”, “tuberculosis” and “review”; individual publications noted to have >224 cases were then examined for the actual number of subjects with both TB and pregnancy; two publications had greater numbers [33, 34]). By including the 3 months before and after pregnancy, any bias due to an adverse outcome of pregnancy or a woman’s decision not to complete a pregnancy has been largely eliminated. This survey excluded any delay due to the administrative process of notification of tuberculosis in the pregnant population (a possible explanation for the data in reference 21), having access to the primary clinical record. Despite the clinics having different numbers of patients with tuberculosis and different health systems, a consistent finding has been that tuberculosis is more likely to be diagnosed in the postpartum period than during pregnancy itself.

This was an observational study using data obtained during normal clinical practice. As such, it was dependent on the accuracy of the medical record made by the clinical physician and ignores any bias due to access to health care. Use of the national crude birth rate might account for the significant differences among the clinics between the predicted and the observed numbers of those who had been pregnant and had a history of tuberculosis. For instance, the national crude birth rate in the United Kingdom was 12 per 1,000, but was 18 per 1,000 in the location of the UK clinic. Moreover, the birth rate is higher in those who have recently arrived in the UK and the incidence of tuberculosis is also higher in these populations. Follow-up of those who have completed treatment for tuberculosis is likely uncommon (follow-up is not recommended in the NICE guidelines relevant to this survey [35]) and the data for being pregnant within 3 months of completing treatment could have been underestimated.

### Generalisability

The study was undertaken in European countries with a high (Belarus, Ukraine), medium (Serbia, Slovakia, UK) and low (Italy, Belgium) incidence of tuberculosis. Different health systems cover the various countries, ranging from free universal access in the UK to one where the majority pay for health insurance in the USA, where the percentage of contribution to health spending ranges from 96.9 to 45 % respectively [36]. This suggests that there is a general delay in the diagnosis of tuberculosis in pregnancy which is unrelated to incidence and health care system.

## Conclusions

There remains a delay in the diagnosis of tuberculosis in pregnancy. Screening for tuberculosis and encouraging antenatal care in high-risk populations may both be important to reduce morbidity due to tuberculosis. Although tuberculosis may be more common in pregnancy, our data do not support a higher rate than expected from the crude birth rate.
